# Genome-wide characterization and expression profiling of the *TGA* gene family in sweet orange (*Citrus sinensis*) reveal *CsTGA7* responses to multiple phytohormones and abiotic stresses

**DOI:** 10.3389/fpls.2025.1530242

**Published:** 2025-02-25

**Authors:** Min Wang, Yue Ma, Yu-Xin Qiu, Si-Si Long, Wen-Shan Dai

**Affiliations:** ^1^ China-USA Citrus Huanglongbing Joint Laboratory, National Navel Orange Engineering Research Center, College of Life Sciences, Gannan Normal University, Ganzhou, Jiangxi, China; ^2^ Jiangxi Provincial Key Laboratory of Pest and Disease Control of Featured Horticultural Plants, Gannan Normal University, Ganzhou, Jiangxi, China

**Keywords:** *Citrus sinensis*, TGA transcription factor, genome-wide identification, abiotic stress, expression pattern

## Abstract

Citrus is widely recognized as one of the most economically important fruit crops worldwide. However, citrus growth is frequently hindered by external environmental stresses, which severely limit its development and yield. The TGA (TGACG motif-binding factor) transcription factors (TFs) are members of the bZIP family and play essential roles in plant defense responses and organ development. Nevertheless, the systematic identification and functional analysis of the TGA family in citrus remains unreported. In this study, genome-wide analysis identified a total of seven CsTGA TFs in *Citrus sinensis*, which were classified into five subgroups. Phylogenetic and syntenic analysis revealed that the *CsTGA* genes are highly conserved, with no tandem or segmental duplication events among family members. Promoter sequence analysis identified numerous *cis*-acting elements associated with transcriptional regulation, phytohormone response, and environmental adaptation in the promoters of *CsTGA* genes. The expression patterns under five phytohormones and three abiotic stresses demonstrated significant responses of multiple *CsTGA* genes under various forms of adversity. Among all tested treatments, *CsTGA7* showed the most robust response to multiple stresses. Tissue-specific expression pattern analysis revealed potential functional biases among *CsTGA* genes. In-depth analysis showed that CsTGA7 localized in the nucleus and possessed transcriptional activation activity, consistent with the typical characteristic of transcriptional regulators. In summary, our research systematically investigated the genomic signature of the TGA family in *C*. *sinensis* and unearthed *CsTGA7* with potential functions in phytohormone signaling transduction and abiotic stress responses. Our study establishes a basis for further exploration of the function of *CsTGA* genes under abiotic stress.

## Introduction

Citrus is one of the most commercially valuable fruits globally, ranking first in both yield and cultivation area among fruit crops. China is the world’s largest citrus producer, with major production areas distributed across southern provinces, including Jiangxi, Hunan, Hubei, Guangxi, Yunnan, and Zhejiang (Huang et al., 2023). According to statistics, the total citrus production in China reached 60,038,900 tons in 2022, making great contributions to increasing the income of farmers, promoting the employment of residents, and boosting rural revitalization. Sweet orange (*Citrus sinensis*), a perennial fruit of the genus *Citrus* within the Rutaceae family, is characterized by its bright color, strong aroma, and rich flavor (Mahato et al., 2017; Liao et al., 2023). As the most dominant fresh citrus variety, sweet oranges generate substantial market demand due to their unique flavor, rich nutritional value, and significant economic value (Xu et al., 2013). However, sweet oranges exhibit relatively low environmental adaptability and frequently suffer from various stresses during their growth and development, such as low temperature, salinity, drought, pests, and diseases, which seriously restricts the healthy and sustainable development of the sweet orange industry (Geng and Liu, 2018). Therefore, breeding sweet orange varieties with stress resistance is a key priority in current citrus research. As perennial woody plants, citrus fruit trees possess long growth cycles and unique reproductive barriers caused by polyembryony, making germplasm innovation through traditional crossbreeding approaches highly challenging (Wang et al., 2022). However, the advent of advanced genetic engineering technologies provides an effective approach for citrus genetic improvement and breeding of stress-resistant varieties. Moreover, identifying key resistance genes in sweet orange under stress conditions is essential for genetic improvement through genetic engineering.

TGA (TGACG motif-binding factor) transcription factors (TFs), a Group D subfamily of the basic leucine zipper (bZIP) TF family, bind to the TGACG sequence in the promoters of target genes to regulate their transcript levels. They play essential roles in plant biological processes, including stress response and organ development (Wang et al., 2020; Yildiz et al., 2023; Gutsche et al., 2023). The tobacco TGA1a was the first TGA TF isolated and characterized from plants, which could bind to the activating sequence 1 (as-1) motif of the virus (Jupin and Chua, 1996). Subsequently, the TGA family has been identified and characterized in various plant species, such as Arabidopsis (*Arabidopsis thaliana*), rice (*Oryza sativa*), soybean (*Glycine max*), peanut (*Arachis hypogaea*), papaya (*Carica papaya*), and sugarcane (*Saccharum* sp*ontaneum*) (Idrovo Espín et al., 2012; Li et al., 2019a; Han et al., 2022; Li et al., 2022; Zhao et al., 2023a; Zhong et al., 2023). Previous studies have shown that the TGA proteins are crucial regulators of plant growth, development, and physiological processes, playing vital roles in phytohormone signaling, stress and disease resistance, as well as organ development (Kobayashi et al., 2024). For example, 10 members of the AtTGA TFs were identified in Arabidopsis, with 7 involved in stress responses and 3 regulating floral organ development (Gatz, 2013). To date, studies have revealed that the number of TGA members in terrestrial plants ranges from 6 in papaya (*Carica papaya*) to 44 in sugarcane (*Saccharum officinarum*), with 10 in Arabidopsis, 16 in rice, 20 in peanut, and 25 in soybean (Idrovo Espín et al., 2012; Gatz, 2013; Chern et al., 2014; Li et al., 2019a; Zhao et al., 2023a; Zhong et al., 2023). The 10 AtTGA members identified in Arabidopsis are categorized into 5 evolutionary subgroups based on sequence similarity and conserved structural domains (Gatz, 2013). Typical TGA proteins contain a conserved leucine-zipper region at the N-terminus and a glutamine-rich basic region at the C-terminus, which serves as the DNA-binding site for the TGA TFs (Tomaž et al., 2022). Studies have shown that the N-terminal leucine-zipper domain of TGA TF is essential for dimerization, while the C-terminal basic region is critical for binding to *cis*-acting elements within the promoters of target genes and executing transcriptional activation (Lu et al., 2024).

Numerous studies have demonstrated that TGA TFs play influential roles in plant responses to abiotic stresses. Overexpression of *MhTGA2* from *Malus hupehensis* significantly upregulated osmotic stress-related genes and enhanced salt and osmotic stress tolerance of transgenic tobacco and apple (Du et al., 2013). *GmTGA13* maintained normal growth of soybean under high salt conditions by reducing Na^+^ uptake and increasing K^+^ and Ca^2+^ accumulation (Ke et al., 2021). MeTGA2 from *Manihot esculenta* interacts with the glutaredoxin MeGRXC3 and regulates the expression of the peroxidase *MeCAT7* through the MeTGA2-MeMYB63 pathway, which participates in ROS homeostasis and stomatal movement in response to drought stress (Guo et al., 2022). Overexpression of *AhTGA11* in *A*. *hypogaea* effectively increased the antioxidant enzyme activities in transgenic *Arabidopsis* under low temperature and attenuated the freezing damage (Zhong et al., 2023). In summary, TGA TFs play a crucial role in plant stress adaptation, but up to now, there is no report on the systematic identification of the TGA family in citrus, and the functional roles of TGA TFs in citrus under abiotic stress remain poorly understood.

In this study, a systematic identification of the TGA family was performed in *C. sinensis*. Phylogenetic relationships, chromosomal distributions, gene duplications, and syntenic relationships of *CsTGA* genes were comprehensively analyzed. Furthermore, the expression patterns of *CsTGA* genes in response to five phytohormone and three abiotic stress treatments were investigated. *CsTGA7*, which displayed the most robust response to all tested treatments, was identified. Further analysis demonstrated that CsTGA7 is a nuclear-localized protein with transcriptional activation activity, consistent with its characterization as a TF. This study holds both theoretical and practical significance, providing genetic resources for the genetic improvement and breeding of sweet orange, as well as a scientific foundation for elucidating the mechanism of TGA family function under abiotic stress in *C. sinensis*.

## Materials and methods

### Plant cultivation and multiple stress treatments

Seedlings of sweet orange (*C*. *sinensis*) were cultivated in a growth chamber at 25°C under a 16h light/8h dark photoperiod. After approximately 3 months, the seedlings were subjected to different phytohormone or abiotic stress treatments.

For phytohormone treatments, concentrations of phytohormones were determined based on previous studies (Gong et al., 2014; Dai et al., 2018; Wang et al., 2019; Ming et al., 2021; Murugan et al., 2023). Briefly, five common phytohormones, including 100 mM abscisic acid (ABA), 20 mg/L ethrel (ETH), 5 mg/L gibberellin (GA), 200 μM jasmonic acid (JA), and 500 μM salicylic acid (SA), were applied through foliar spray and soil irrigation to sweet orange seedlings. Leaves were collected at designated time points. For abiotic stress treatments, sweet orange seedlings were treated with low temperature (4°C), 300 mM NaCl, or dehydration. For dehydration treatment, seedlings were carefully removed from pots, cleared of root-attached soil, and placed on filter paper to dehydrate at room temperature. Samples were collected at the corresponding time points following each treatment and stored at -80°C. For tissue-specific expression pattern analysis, sweet orange seedlings were carefully excavated from pots, and the adhering soil was gently cleared. Roots, stems, and leaves were precisely dissected, immediately frozen in liquid nitrogen, and stored at −80°C. At least 10 sweet orange seedlings were used for each treatment, and all samples were preserved for RNA extraction and gene expression analysis.

### Identification of *CsTGA* family members in *C*. *sinensis*

To identify *TGA* genes in *C*. *sinensis*, the 10 AtTGA protein sequences of *Arabidopsis* were downloaded as query sequences from the Arabidopsis Information Resource (TAIR) database (https://www.arabidopsis.org/). BLASTP search was performed against the Citrus Pan-genome to Breeding Database (*Citrus sinensis* v2.0) (http://citrus.hzau.edu.cn/index.php) using an *E*-value threshold of 1e^−5^ to identify CsTGA members. All identified protein sequences were further submitted to the online databases CDD (http://www.ncbi.nlm.nih.gov/Structure/cdd/wrpsb.cgi) and SMART (http://smart.embl-heidelberg.de/) to confirm the presence of the complete bZIP and DOG1 domains (Letunic et al., 2020; Lu et al., 2020). After eliminating sequences containing incomplete conserved domains, all non-redundant proteins corresponding to the longest transcript isoforms were retained as the final set of CsTGA proteins. ExPASy (https://web.expasy.org/protparam/) was used to calculate the theoretical isoelectric point (pI) and molecular weight (MW) of the TGA proteins (Gasteiger et al., 2005).

### Phylogenetic analysis and sequence alignment

TGA family protein sequences from *Arabidopsis* (*A*. *thaliana*) were obtained from the TAIR website (https://www.arabidopsis.org/), while TGA family protein sequences from rice (*O*. *sativa*) were downloaded from the NCBI website (https://www.ncbi.nlm.nih.gov/). Additionally, TGA family protein sequences from common bean (*Phaseolus vulgaris*), sorghum (*Sorghum bicolor*), and soybean (*G*. *max*) were acquired from the Phytozome website (https://phytozome-next.jgi.doe.gov/) (Liu et al., 2023). Accession numbers for all TGA proteins mentioned above are listed in **Supplementary Table S1**. The phylogenetic analysis was constructed using the MEGA software based on the amino acid (aa) sequences of TGA proteins, using the neighbor-joining (NJ) method (Bootstrap = 1000). The obtained phylogenetic tree was landscaped and presented using the online tool iTOL (https://itol.embl.de/). Multiple sequence alignment of CsTGA proteins was performed using ClustalW and visualized by the Jalview software (Larkin et al., 2007; Waterhouse et al., 2009).

### Conserved motif and gene structure analysis

Conserved motifs of CsTGA proteins were characterized using MEME software with a maximum of six motifs and an optimal motif width ranging from 6 to 60 aa residues (Bailey et al., 2015). The “Gene Structure View” module in TBtools software (version 2.096) was used to visualize the gene structures of the *CsTGA* members based on the genomic GFF file of *C*. *sinensis* (Chen et al., 2020). The tertiary structures of CsTGA proteins were predicted using AlphaFold3 (https://alphafoldserver.com/) and visualized using PyMOL software (Abramson et al., 2024).

### Chromosome localization, gene duplication and synteny analysis

Based on the chromosomal position information from the GFF annotation file, the *CsTGA* genes were precisely mapped onto nine chromosomes in ascending order of physical positions (bp). The MCScanX software was used for intra- and inter-species collinearity analysis of TGA proteins with an E-value threshold of 1e^−5^, a maximum gap size of 25, and a minimum match score of 20 (Wang et al., 2012). Visualization was accomplished using the “Amazing Super Circo” and “Multiple Synteny Plot” modules in the TBtools software (version 2.096), respectively (Chen et al., 2020).

### Promoter *cis*-acting elements analysis

The 2.5 kb upstream promoter sequences of the seven *CsTGA* genes were extracted from the *C*. *sinensis* genome using the “GFF3 Sequence Extract” module in TBtools software (version 2.096). Promoter cis-acting elements were analyzed using the online platform PlantCARE (http://bioinformatics.psb.ugent.be/webtools/plantcare/html/) (Lescot et al., 2002). Finally, the identified *cis*-acting elements were landscaped and displayed using the “Sample Biosequence View” module in TBtools.

### Quantitative real-time PCR analysis

RNA extraction was performed using leaves collected and stored at −80°C. Total RNA was extracted using the OminiPlant RNA Kit (CWBIO, China), and cDNA was synthesized using the PrimeScript™ RT Kit and the gDNA Eraser (Takara, Japan), following the manufacturer’s instructions. “Batch qPCR primer design” module of TBtools software (version 2.096) was used to design specific quantitative primers for the *CsTGA* genes, and the “Primer Check” module was used to assess primer quality, as detailed in **Supplementary Table S2** (Chen et al., 2020). The *CsActin* gene was selected as an internal control, 2×TSINGKE^®^ Master qPCR Mix (TSINGKE, China) was employed to construct the reaction system for gene expression analysis. The QuantStudio 5 Applied BioSystem (Thermo Fisher Scientific, Waltham, MA, USA) was utilized for fluorescence detection during qRT-PCR. Each sample was analyzed with three biological replicates and three technical replicates, and relative expression levels were calculated using the 2^-ΔΔCt^ method.

### Subcellular localization assay

The full-length (FL) coding sequence (CDS, 1530 bp) of *CsTGA7* was amplified using cDNA of sweet orange as a template and cloned into the pBI121-EGFP binary expression vector driven by the Cauliflower mosaic virus 35S (CaMV 35S) promoter with *Xba* I and *Xma* I restriction sites added for insertion. The recombinant plasmid and the empty vector were independently transformed into *Agrobacterium tumefaciens* strain GV3101. Subsequently, the *Agrobacteria* suspensions were transiently infiltrated into tobacco (*Nicotiana benthamiana*) leaves using *A*. *tumefaciens*-mediated transformation as previously described (Dai et al., 2023). A *VirD2NLS* gene fused to mCherry was used as a nuclear marker. Tobacco plants were cultured in the dark for 12h, followed by 3 days in a light incubator (16h of light/8h of dark) at 21°C. The infiltrated tobacco leaves were transferred to a confocal laser scanning microscope (Leica TCS SP8, Germany) system to observe green fluorescence (GFP) and red fluorescence (mCherry) signals.

### Transcriptional activation activity analysis

The CDS of CsTGA7 (1–1527 bp), along with its N-terminal (1–840 bp) and C-terminal (841–1527 bp) truncated fragments, were cloned into the *EcoR* I and *BamH* I restriction sites of the pGBKT7 vector. The three recombinant plasmids were independently transformed into Y2HGold yeast cells according to the manufacturer’s instructions (Matchmaker^®^ Gold Yeast Two-Hybrid Library Screening System, Takara) and cultured on SD/-Trp selective medium. The pGBKT7-53 and pGBKT7-lam were simultaneously transformed as positive and negative controls, respectively. After 3 days, the positive transformants were cultured on SD/-Trp and SD/-Trp/-His/-Ade+X-α-gal (Sigma-Aldrich, USA) selective medium to detect their transcriptional activation activity.

## Results

### Genome-wide identification and phylogenetic analysis of the *CsTGA* gene family

A BLASTP search was performed against the sweet orange protein database utilizing the aa sequence of AtTGA from *A*. *thaliana* as queries. Initially, 36 CsTGA proteins were identified, and seven non-redundant CsTGA members with complete bZIP and DOG domains were retained for further analysis. Detailed information on *CsTGA* genes is provided in **Supplementary Table S3**. The CsTGA members were named from *CsTGA1* to *CsTGA7* following the nomenclature proposed by Soranzo et al. (2004). The open reading frames (ORFs) of the seven *CsTGA* genes ranged from 1080 bp (*CsTGA1*) to 1533 bp (*CsTGA7*) in length, encoding proteins ranging from 359 to 510 aa. Their molecular weights (mW) ranged from 40.82 kDa to 56.37 kDa, and predicted isoelectric point (pI) ranged from 6.28 (CsTGA3) to 7.37 (CsTGA5). Notably, the pI values of most CsTGA proteins, except for CsTGA3 and CsTGA4, were higher than 7, suggesting that most CsTGA proteins were rich in basic aa residues (**Supplementary Table S3**).

**Figure 1:**
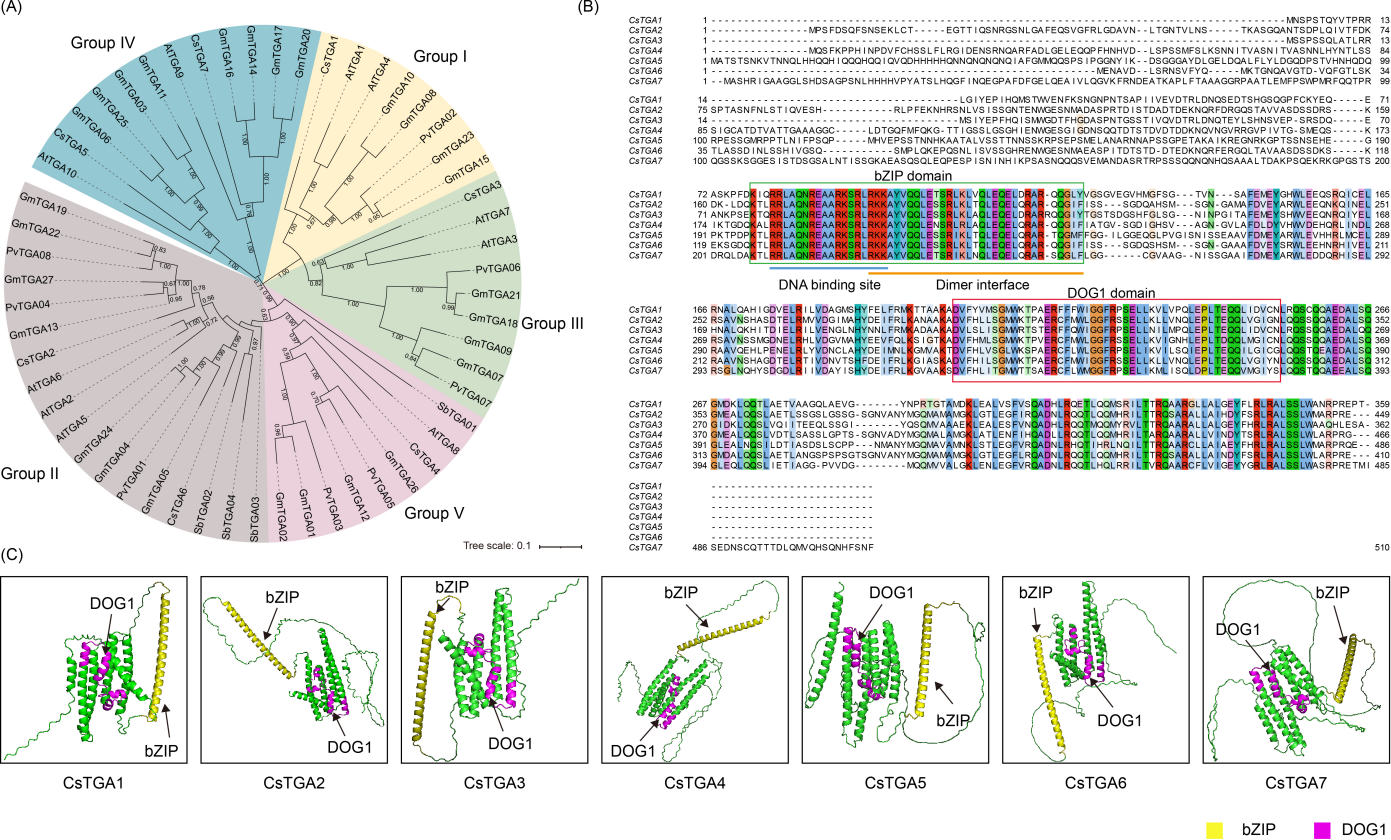
Identification of *TGA* genes in *C sinensis*. **(A)** Phylogenetic analysis of TGA genes in sweet orange (Cs, *C*. *sinensis*), Arabidopsis (At, *A*. *thaliana*), rice (Os, *O*. *sativa*), common bean (Pv, *P*. *vulgaris*), sorghum (Sb, *S*. *bicolor*), and soybean (Gm, *G*. *max*). The phylogenetic tree was constructed using MEGA with the NJ method and 1,000 bootstrap replicates. Based on the bootstrap values and evolutionary distances, the tree was clustered into five subgroups (Groups I–V). **(B)** Multiple sequence alignment of the CsTGA proteins. **(C)** Representative predicted 3D protein structures for each of seven CsTGA proteins, performed by AlphaFold3. Yellow structure represents the bZIP domain and plum structure represents the DOG1 domain.

To further investigate the evolutionary relationships of CsTGA proteins, the phylogenetic trees were constructed for CsTGA (*C*. *sinensis*, 7), AtTGA (*A*. *thaliana*, 10), PvTGA (*P*. *vulgaris*, 8), SbTGA (*S*. *bicolor*, 4), and GmTGA (*G*. *max*, 27) proteins. Based on the AtTGA sequence of the model plant *A. thaliana*, the subgroups were indicated by different colors (**Figure 1A**) (Gatz, 2013). Based on the evolutionary relationships, the 56 TGA proteins from the five species were categorized into five subgroups (Groups I–V), with the seven CsTGA members evenly distributed. Among the five subgroups, Groups I, III, and V individually contained one *CsTGA* gene, namely *CsTGA1*, *CsTGA3*, and *CsTGA4*. Groups II and IV each contained two *CsTGA* genes, which were *CsTGA2*/*CsTGA6* and *CsTGA5*/*CsTGA7*, respectively. Multiple sequence alignment revealed that aa residues in the essential bZIP and DOG1 domains were highly conserved among the CsTGA proteins (**Figure 1B**). In particular, the N-terminus of the bZIP domain contains 18 highly conserved aa residues forming the DNA-binding basic region, while the C-terminus contributes to the dimeric leucine zipper structure. Furthermore, the tertiary structures (3D models) of all CsTGA proteins were predicted using AlphaFold3 and presented in **Figure 1C**. The bZIP domain, responsible for DNA binding, exhibited a characteristic helical structure, while the DOG1 domain, involved in dimerization and regulatory functions, displayed a more complex folding pattern. The structural integrity of these domains underscores their critical roles in the transcriptional regulation and stress responses mediated by CsTGA proteins. Notably, the 3D models also highlighted potential interaction sites and conformational changes that may influence the functional specificity and efficiency of these proteins.

### Conserved domain and gene structure of *CsTGA* genes

**Figure 2:**
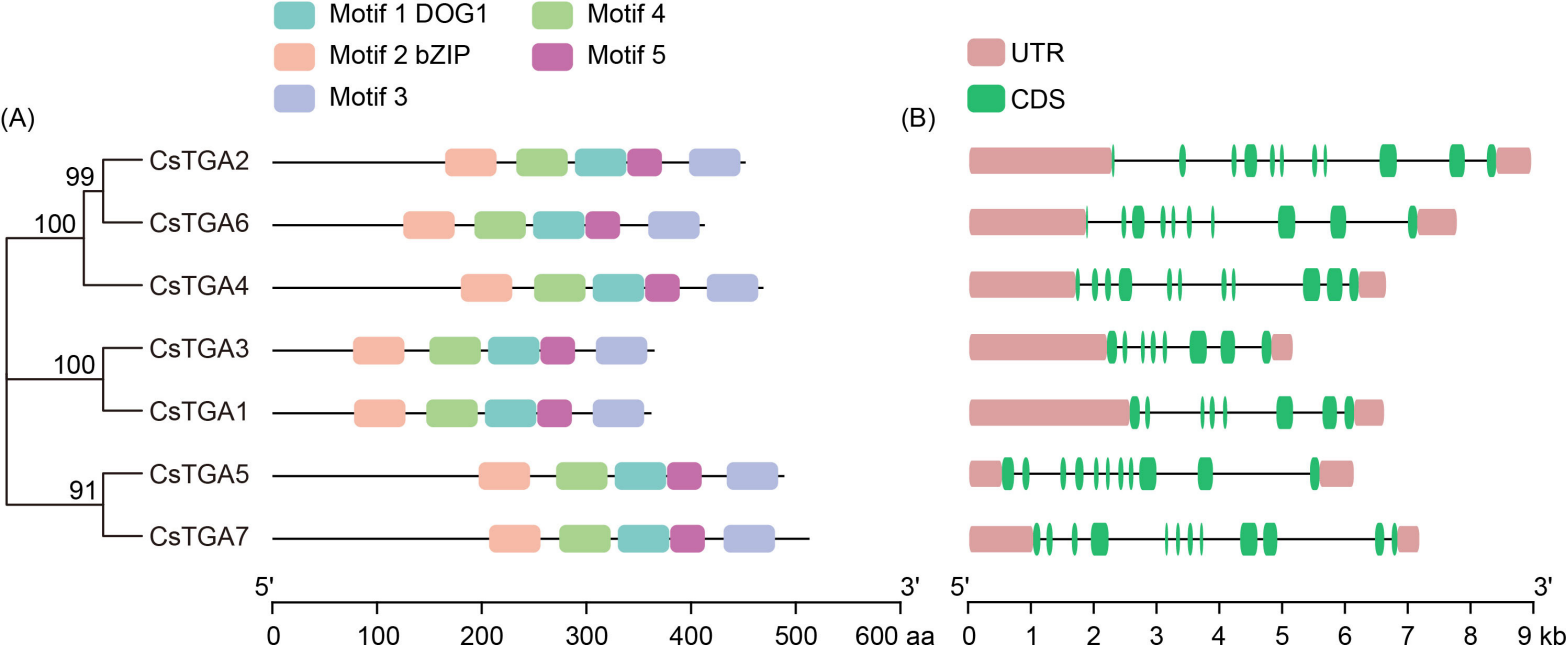
Conserved motif and gene structure analysis of *CsTGA* genes. **(A)** The distribution of five conserved motifs in CsTGA proteins is shown by different colored blocks. The sequences of these conserved motifs are provided in **Supplementary Table S4**. **(B)** Exon/intron distributions of *CsTGA* genes. The exons and introns are represented by green boxes and black lines, respectively. Pink boxes indicate the upstream and/or downstream untranslated regions.

To reveal the structural features of the CsTGA family, the conserved motifs of the CsTGA proteins were first analyzed. A total of five conserved motifs were detected, ranging in length from 34 to 50 aa (**Supplementary Table S4**). Among them, motif 1 was annotated as the DOG1 domain, which has been demonstrated to be associated with phytohormone response and seed dormancy regulation (Iwasaki et al., 2022). Motif 2 was annotated as the bZIP domain, which is normally involved in nuclear localization, DNA binding, and dimer formation of TGA proteins (Dröge-Laser et al., 2018). The conserved DOG1 and bZIP domains were present in all CsTGA proteins (**Figure 2A**). A multiple sequence alignment of the core DOG1 and bZIP domains of CsTGA proteins is shown in **Supplementary Figure S1**. It is noteworthy that the positions of conserved motifs were comparable among the closely related CsTGA family members. For example, the relative positions of five conserved motifs in CsTGA5 and CsTGA7 (Group IV) were nearly identical (**Figure 2A**). To further elucidate the structural diversity of *CsTGA* genes, the distribution of gene exon/intron structures was analyzed. The results showed that most CsTGA genes contained approximately 10 exons, with the exception of *CsTGA1* and *CsTGA3*, which had only 8 exons (**Figure 2B**).

### Chromosomal location, gene duplication, and syntenic analysis of *CsTGA* genes

**Figure 3:**
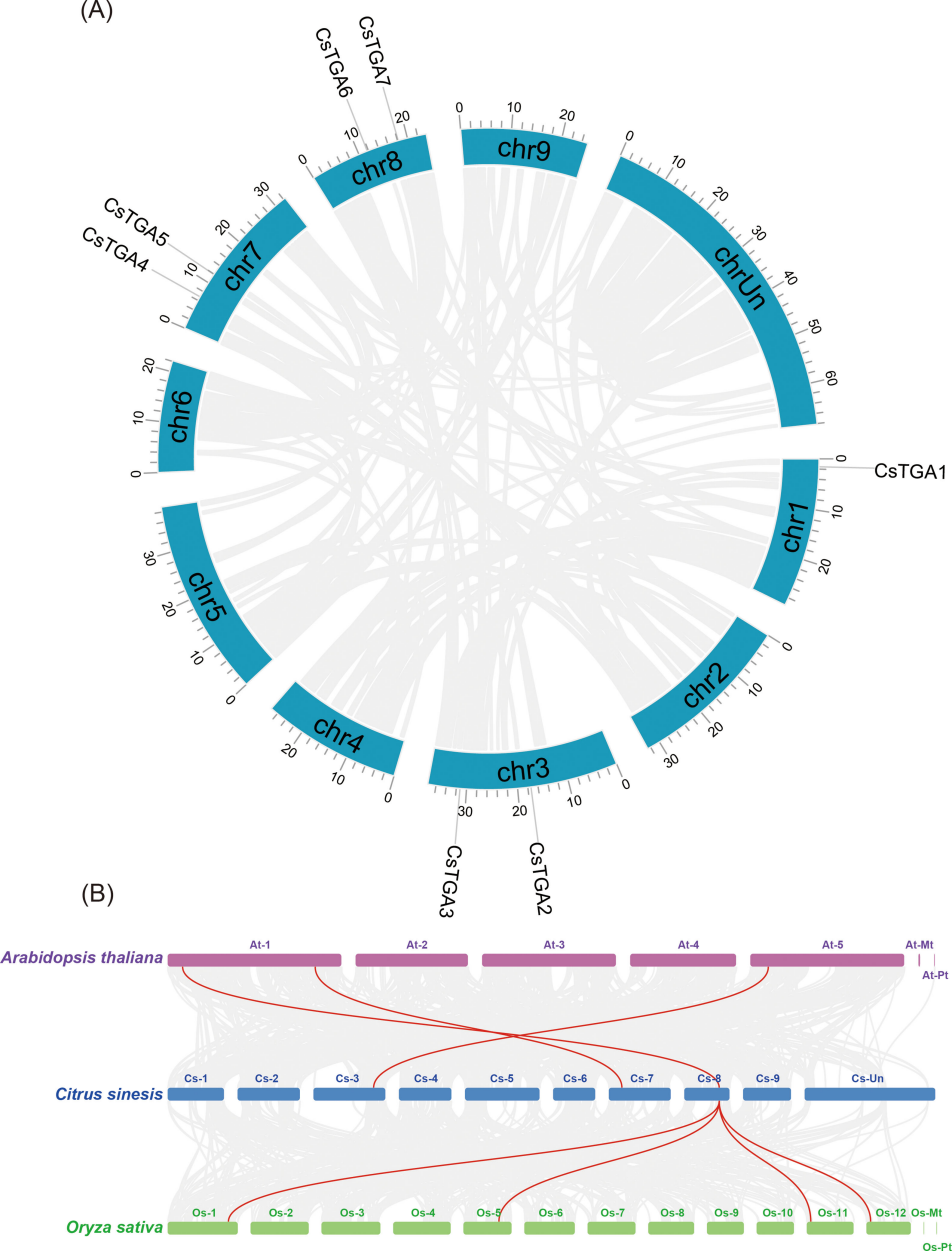
Duplication and synteny analysis of *TGA* genes. **(A)** Chromosomal location of *CsTGA* genes. Blue columns represent chromosomes with the chromosome numbers placed in the middle and the gene ID outside the plot. **(B)** Synteny relationships of *TGA* genes among *C. sinensis, A. thaliana*, and *O*. *sativa*. Horizontal columns represent chromosomes with chromosome numbers placed in the middle. Gray lines indicate the collinear blocks between genome pairs, and syntenic *CsTGA* genes are linked by red lines.

Chromosomal localization of CsTGA genes was mapped using the TBtools software. The seven *CsTGA* genes were unevenly distributed across four sweet orange chromosomes (**Figure 3A**). Chromosome 1 harbored one *CsTGA* gene, while chromosomes 3, 7, and 8 each contained two *CsTGA* genes. During evolution, chromosomal amplification could undergo in the genome through various manners, such as segmental duplication, tandem duplication, or genome-wide duplication, leading to gene family expansion. Duplicated genes often exhibit functional overlap or redundancy (Clark, 2023). Therefore, gene duplication events provide valuable insights into the evolution and function of the gene families. Interestingly, the intraspecific synteny analysis showed no tandem or segmental duplication events among *CsTGA* members in the *C*. *sinensis* genome, suggesting that the evolution of *CsTGA* gene families may have originated from other duplication events.

To further investigate the evolutionary relationships of the *CsTGA* genes in different species, we selected the dicot model plant *Arabidopsis* (*A*. *thaliana*) and the monocot model plant rice (*Oryza sativa subsp*. *japonica*) as the reference genomes and generated genomic collinearity plots between *CsTGA*, *AtTGA*, and *OsTGA* genes. The analysis identified three syntenic pairs between *CsTGA* genes and *AtTGA* genes, and four syntenic pairs between *CsTGA* genes and *OsTGA* genes, suggesting that the *CsTGA* genes are more closely related to the *OsTGA* genes in monocots during evolution (**Figure 3B**).

### *cis*-acting elements within the promoter regions of the *CsTGA* genes

**Figure 4:**
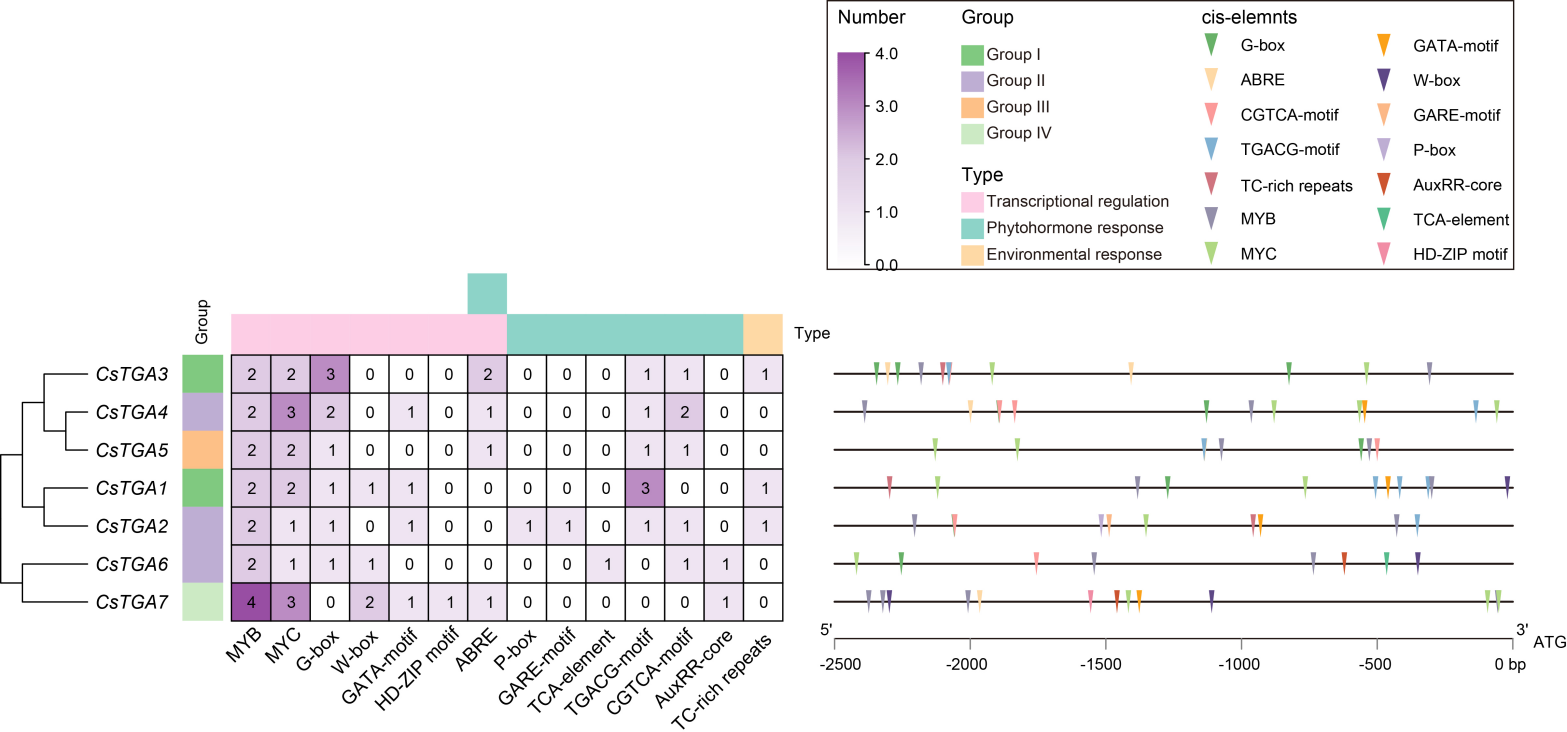
*cis*-acting element analysis in the promoters of *CsTGA* genes. *cis*-acting elements were identified in the 2.5 kb upstream promoter regions of *CsTGA* genes. The numbers in the heatmap represent the count of corresponding *cis*-acting elements. A color scale is displayed vertically at the top right of the diagram. Different colored triangles represent different *cis*-acting elements. Detailed information of sequence and position of these *cis*-acting elements is described in **Supplementary Table S4**.

Promoters are situated upstream of the transcription start site (TSS) of genes, and the presence of extensive *cis*-acting elements in them is critical for the regulation of gene expression (Shrestha et al., 2018). In order to comprehend the genetic processes and regulatory networks of CsTGA genes, the *cis*-elements in the promoter regions of each *CsTGA* were examined. A large number of *cis*-acting elements associated with transcriptional regulation were identified in the promoters of *CsTGA* genes (**Figure 4**, **Supplementary Table S5**). For instance, MYB TF binding sites (16), MYC TF binding sites (14), and WRKY TF binding sites (4). Additionally, a substantial number of *cis*-acting elements related to phytohormone response and environmental stress were found in the promoters of the *CsTGA* genes, including P-box and GARE-motif (gibberellin-responsive element), TCA-element (salicylic acid-responsive element), TGACG-motif and CGTCA-motif (jasmonic acid-responsive element), AuxRR-core (auxin-responsive element), and TC-rich repeats (defense- and stress-responsive element). In summary, these findings suggest that the *CsTGA* genes may be involved in transcriptional regulation, plant hormone signaling transduction, and environmental stress response.

### Expression pattern analysis of *CsTGA* genes under multiple phytohormone treatments

**Figure 5:**
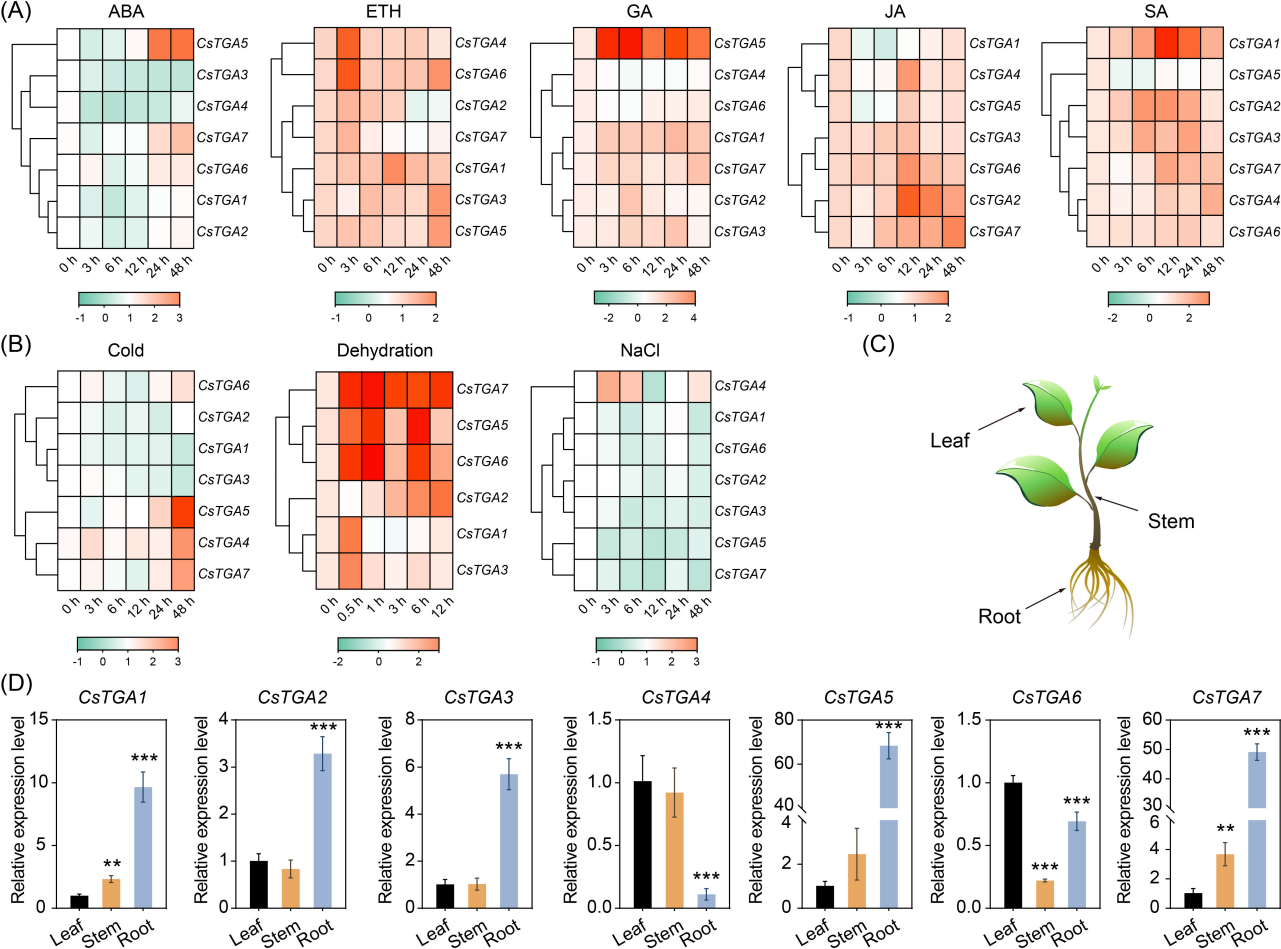
Expression profiles of *CsTGA* genes. **(A)** Expression profiles of *CsTGA* genes under multiple phytohormone treatments. Expression analysis was performed in leaves of *C*. *sinensis* at different time points (0, 3, 6 12, 24, and 48 h after treatments). **(B)** Expression profiles of *CsTGA* genes under multiple abiotic stresses. Expression analysis was performed in leaves of *C*. *sinensis* at different time points (0, 3, 6, 12, 24, and 48 h after NaCl and cold treatments; 0, 0.5, 1, 3, 6, and 12 h after dehydration treatment). The qRT-PCR results of *CsTGA* genes were normalized using log_2_ transformation. The heatmap constructed by TBtools software. A color scale is displayed horizontally at the bottom of the diagram. **(C)** Schematic diagram of different tissues sampled from sweet orange seedlings. **(D)** Tissue-specific expression patterns of *CsTGA* genes. The asterisk indicates the significant difference between the tested group and the leaf group based on a Tukey’s test (**p < 0.01, ***p < 0.001).

Promoter analysis revealed significant enrichment of *cis*-acting elements related to phytohormone response in the promoter region of the *CsTGA* genes, such as P-box, GARE-motif, and TCA-element, indicating their potential roles in response to phytohormones in *C*. *sinensis*. In order to deeply investigate the response patterns of *CsTGA* genes to phytohormones, qRT-PCR was employed to analyze the expression patterns of seven *CsTGA* genes following the treatment with five key phytohormones: ABA, ETH, GA, JA, and SA. Treatment with the five phytohormones induced distinct expression patterns across multiple *CsTGA* genes (**Figure 5A**). Under ABA treatment, most *CsTGA* genes were significantly downregulated, except for *CsTGA5* and *CsTGA7*, which exhibited pronounced upregulation. Under ETH treatment, only *CsTGA2* and *CsTGA7* were significantly downregulated, while the remaining *CsTGA* genes showed remarkable upregulation. Following GA treatment, most *CsTGA* genes displayed significant upregulation, whereas only *CsTGA4* and *CsTGA6* were markedly downregulated. Notably, the expression level of *CsTGA5* increased by 180-fold at 6h post-treatment, indicating a rapid and robust response to GA. Following JA treatment, the *CsTGA2*, *CsTGA4*, *CsTGA6*, and *CsTGA7* were upregulated, while *CsTGA1* and *CsTGA5* were downregulated. No significant change in expression was observed for *CsTGA3*. Under SA treatment, the majority of *CsTGA* genes were significantly upregulated, whereas only *CsTGA5* was downregulated (**Figure 5A**, **Supplementary Table S6**).

### Expression pattern analysis of *CsTGA* genes under multiple abiotic stresses

To better understand the roles of *CsTGA* genes in abiotic stress response in *C*. *sinensis*, qRT-PCR was performed to analyze the response patterns of seven *CsTGA* genes under three general abiotic stresses: low temperature (cold), dehydration, and salt stress (NaCl) (**Figure 5B**). After cold treatment, *CsTGA1*, *CsTGA2*, *CsTGA3*, and *CsTGA6* were significantly downregulated, whereas *CsTGA4*, *CsTGA5*, and *CsTGA7* were markedly upregulated. Following dehydration treatment, all *CsTGA* genes were differentially upregulated, with most *CsTGA* genes exhibiting significant upregulation at 0.5h post-treatment, indicating a rapid response of *CsTGA* genes to dehydration stress. Notably, the expression level of *CsTGA4* was undetectable. In contrast, under salt treatment, only *CsTGA4* was significantly upregulated, while all other *CsTGA* genes were extremely downregulated (**Figure 5B**, **Supplementary Table S7**).

### Tissue-specific gene expression pattern analysis of *CsTGA* genes

Tissue-specific gene expression provides critical insights into functional roles and regulatory mechanisms across different plant tissues. To better understand the biological functions and potential regulatory networks of *CsTGA* genes in sweet orange, we analyzed their expression patterns across various tissues (**Figures 5C, D**). *CsTGA1* and *CsTGA7* exhibited similar tissue-specific expression profiles, with the highest expression in roots, followed by stems, and the lowest expression in leaves, suggesting their potential roles in root and stem physiology, possibly contributing to coordinated growth and development in both underground and aboveground tissues of *C*. *sinensis*. Similarly, *CsTGA2*, *CsTGA3*, and *CsTGA5* displayed comparable tissue-specific expression patterns, with high transcript levels in roots and no significant differences between leaves and stems, implying their potential roles in root-specific processes, such as root development, nutrient uptake, or responses to soil-related stresses. In contrast, *CsTGA4* exhibited no significant difference in expression between leaves and stems but was markedly downregulated in roots, indicating its primary function in aboveground tissues, potentially involved in photosynthesis, metabolic regulation, or stem development. Finally, *CsTGA6* showed the lowest relative expression in stems, followed by roots, and the highest expression in leaves, indicating a predominant role in leaf-specific processes, such as leaf development. In summary, the tissue-specific expression profiles of *CsTGA* genes in *C*. *sinensis* reveal significant differences, indicating functional diversification within the *CsTGA* gene family.

### Screening of *CsTGA* genes in response to multiple treatments

**Figure 6:**
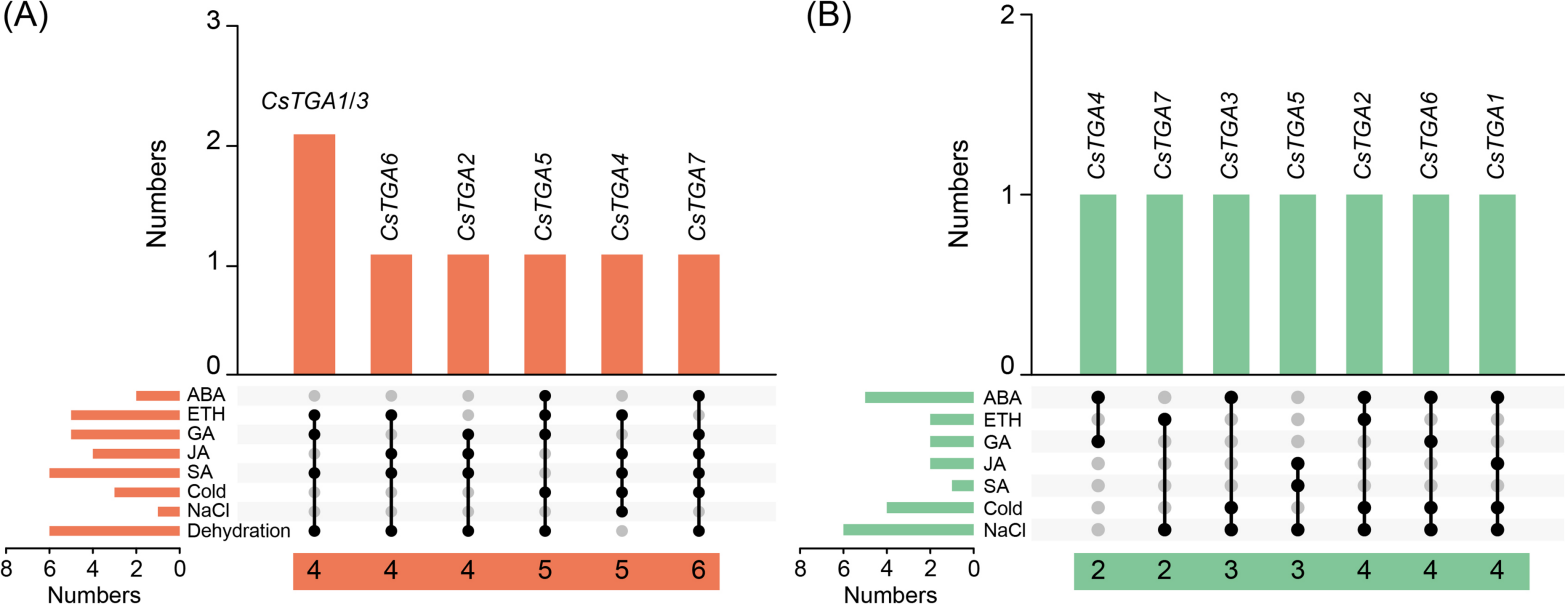
Venn diagram of *CsTGA* genes under phytohormone and abiotic stress treatments. **(A)** Overlap of *CsTGA* genes upregulated under phytohormone and abiotic stress treatments. Orange columns represent the number of overlapping treatments with upregulated *CsTGA* genes. The orange columns in the lower left corner represent the number of upregulated *CsTGA* genes under each treatment. Numbers in the horizontal orange blocks at the bottom indicate the number of treatments to which the individual genes responded. Black circles connected by lines represent the overlapping treatments. **(B)** Overlap of *CsTGA* genes downregulated under phytohormone and abiotic stress treatments. Viridescent columns represent the number of overlapping treatments with downregulated *CsTGA* genes. The viridescent columns in the lower left corner represent the number of downregulated *CsTGA* genes under each treatment. Numbers in the horizontal viridescent blocks at the bottom indicate the number of treatments to which the individual genes responded. Black circles connected by lines represent the overlapping treatments.

To identify *CsTGA* genes with significant responses to multiple treatments, we constructed Venn diagrams of upregulated and downregulated *CsTGA* genes based on their expression patterns following phytohormone and abiotic stress treatments, respectively. Among the upregulated genes, four genes (*CsTGA1/2*/*3*/*6*) were able to respond to four different treatments. *CsTGA5* and *CsTGA4* could respond to five different phytohormone and abiotic stress treatments, respectively. Notably, *CsTGA7* was significantly upregulated under all six treatments except ETH and NaCl (**Figure 6A**). Conversely, among the downregulated genes, two genes (*CsTGA4*/*7*) could significantly respond to two different treatments, while two genes (*CsTGA3*/*5*) showed marked responses to three different treatments. In addition, three genes (*CsTGA1*/*2/6*) exhibited an extreme downregulated expression trend in response to four different treatments (**Figure 6B**). In conclusion, *CsTGA7*, which showed the most robust response to various stresses among all tested treatments, was selected as the primary candidate for further investigation.

### CsTGA7 is a typical transcription factor

**Figure 7:**
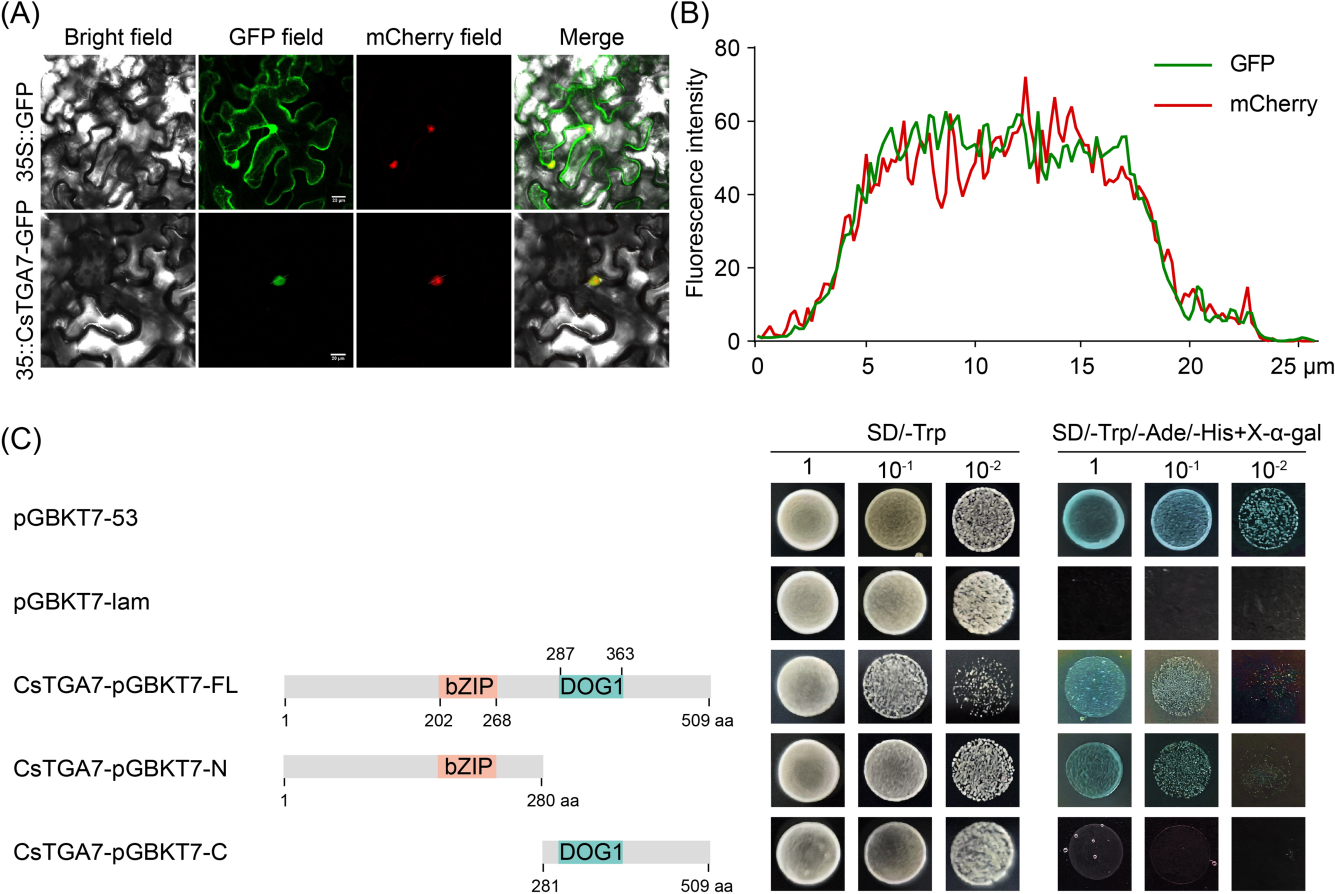
Subcellular localization and transcriptional activation activity of CsTGA7. **(A)** The 35S::GFP and 35S::CsTGA7-GFP constructs were transiently expressed in *N*. *benthamiana* leaves *via A. tumefaciens*-mediated transformation and observed under a confocal laser scanning microscope. Scale bars represent 20 µm. **(B)** Fluorescence intensity profiles of GFP and mCherry at the position shown by the white line in the 35S::CsTGA7-GFP localization image shown in **(A)**. **(C)** Growth of yeast cells co-transformed with different vectors on selective medium. Full-length (CsTGA7-pGBKT7-FL), N-terminus (CsTGA7-pGBKT7-N), and C-terminus (CsTGA7-pGBKT7-C) fragments of *CsTGA7* were introduced in pGBKT7 vector. The numbers above or beneath the bars indicate the positions of amino acid residues.

Transcriptional regulation typically occurs in the nucleus, making nuclear localization a typical feature of most TFs (Dai et al., 2023). To further explore the genetic properties of *CsTGA7*, we fused the FL sequence of *CsTGA7* with green fluorescent protein (GFP) under the control of CaMV 35S promoter and transiently expressed in tobacco leaves via *Agrobacterium*-mediated transformation to observe its subcellular localization. As shown in **Figure 7A**, the green fluorescence in tobacco leaves transformed with the empty vector was distributed throughout the entire cell, including the cell membrane, nucleus, and cytoplasm. In contrast, in tobacco cells expressing the CsTGA7-GFP fusion protein, the GFP signal was specifically localized to the nucleus. Further analysis showed that the fluorescence intensity profile of 35S::CsTGA7-GFP highly overlapped with that of the mCherry marker protein (**Figure 7B**; white lines in **Figure 7A** indicating sampling locations for co-localized fluorescence), demonstrating that CsTGA7 was exclusively distributed in the nucleus.

In addition to nuclear localization, most TFs possess transcriptional activation activity (He et al., 2024). To dissect the functional domains of CsTGA7, we investigated its transcriptional activation activity using a yeast system. The CsTGA7 protein was truncated based on its conserved domains, with the N-terminus containing a bZIP domain and the C-terminus harboring a DOG1 domain (**Figure 7C**). Yeast transformation assays were conducted with constructs encoding either the FL of CsTGA7, its N-terminal region, or its C-terminal region. The results showed that yeast transformants expressing either the FL or N-terminal fragment grew on selective dropout medium (SD/-Trp/-Ade/-His) and turned blue in the presence of X-α-gal, indicating successful transcriptional activation leading to reporter gene expression.In contrast, yeast cells transformed with constructs containing only the C-terminal region of CsTGA7 failed to grow on the same selective medium, demonstrating that CsTGA7 possessed transcriptional activation activity, and that the N-terminus was required for this activity.

## Discussion

TGA TFs, belonging to the bZIP family, represent one of the earliest classes of TFs identified in plants and play vital roles in plant defense responses and organ development. In this study, we excavated seven *CsTGA* genes at the whole-genome level in *C*. *sinensis* and systematically analyzed their gene structures, evolutionary relationships, and expression patterns.

### The characterization and evolution of *CsTGA* genes in *C. sinensis*

Seven CsTGA proteins were identified in the *C*. *sinensis* genome by BLASTP search and HMMER analysis. Phylogenetic analysis revealed that the seven *CsTGA* genes were clustered into five subgroups, with an even distribution among the subgroups (**Figure 1A**). Analysis of conserved domains showed that all CsTGA members contain DOG1 and bZIP domains, with conserved motifs exhibiting similar positions and distributions among closely related CsTGA family members (**Figure 2A**). According to a recent study by Zhang et al. (2024), 56 CsbZIP members were identified in *C*. *sinensis*, and classified into 13 subgroups following the classification of AtbZIP proteins from *A. thaliana*. Seven CsbZIPs and 10 AtbZIPs, belonging to the TGA family, were clustered into subgroup D. This is consistent with our findings, further validating the reliability of our study. Exon-intron structures play critical roles in mRNA precursor splicing and are key determinants of gene function (Tammer et al., 2022). Current research concludes that introns enhance exon recombination, facilitating the formation of new genes and increasing evolutionary potential (Zlotorynski, 2019). In the rice genome, the frequency of intron loss after fragment duplication is higher than that of intron gain (Nuruzzaman et al., 2010). Our study revealed that among the *CsTGA* subfamilies in *C*. *sinensis*, *CsTGA5* and *CsTGA7* of Group IV possessed the highest number of introns, suggesting that *CsTGA* genes in Group IV may represent ancestral genes and occupy an evolutionarily basal position, from which other subgroups have diverged.

Gene duplication events, including tandem duplication, segmental duplication, and whole-genome duplication (WGD), are widely recognized as critical evolutionary forces in species formation, adaptation, and diversification (Clark and Donoghue, 2018). In this study, the 322 Mb genome of *C*. *sinensis* contained only seven *TGA* genes, with no evidence of tandem or segmental duplication events among CsTGA members (**Figure 3A**), suggesting that the *CsTGA* genes in *C*. *sinensis* are highly conserved and may have evolved through other types of duplication events. Interspecies synteny analysis demonstrated stronger syntenic relationships between *CsTGA* and *OsTGA* than between *CsTGA* and *AtTGA* (**Figure 3B**). Similar patterns were observed in synteny analyses of the *DREB* family in Moso bamboo (*Phyllostachys edulis*) (Hu et al., 2023). This observation aligns with the hypothesis that monocots and dicots share common ancestral genes prior to their divergence, further supporting the high conservation of *CsTGA* genes in *C*. *sinensis* and the absence of intraspecies duplication events (Li et al., 2019b) It is noteworthy that *CsTGA7* exhibited the most extensive interspecies collinearity, with one syntenic pair to *AtTGA* and four syntenic pairs to *OsTGA* genes, and contained the highest number of introns (11). These findings suggest that *CsTGA7* of Group IV in *C*. *sinensis* may occupy an anterior position in the evolutionary progression of the *CsTGA* family.

### *CsTGA* genes are involved in phytohormone responses

Phytohormones are naturally occurring organic compounds produced in specific plant tissues, forming complex chemical signaling systems that regulate plant growth, development, and responses to environmental stresses (Ali et al., 2024). Numerous studies have demonstrated that TGA TFs play diverse roles in plant hormone signaling pathways (Zander et al., 2012; Guo et al., 2022; Kim et al., 2022; Zavaliev and Dong, 2024). ABA is a central regulator of plant adaptation to environmental stress, exerting profound effects on dormancy, germination kinetics, and the enhancement of stress response pathways (Sun et al., 2022). In this study, all *CsTGA* genes respond significantly to ABA treatment, with *CsTGA5* and *CsTGA7* displaying pronounced upregulation and the other *CsTGA* genes undergoing notable downregulation (**Figure 5A**). Promoter analysis revealed that most *CsTGA* genes (*CsTGA3*/*4*/*5*/*7*) contain at least one ABRE element in their promoter regions (**Figure 4**). Therefore, we hypothesize that *CsTGA* genes may exert an essential role in ABA-mediated physiological processes in *C*. *sinensis*. Ethylene regulates key processes, including seed germination, flower development, leaf senescence, root growth inhibition, and stress responses (Dubois et al., 2018). Except for *CsTGA2* and *CsTGA7*, which were dramatically downregulated, all *CsTGA* genes were significantly upregulated after ETH treatment, indicating that most *CsTGA* genes respond positively to ethylene. GA regulates key biological processes such as fruit yield, stem elongation, and root development (Binenbaum et al., 2018). We noticed that the expression of *CsTGA2* was significantly upregulated upon GA treatment, and its promoter contained several GA-responsive elements, including P-box and GARE-motif (**Figure 4**), suggesting a potential role of *CsTGA2* in GA signaling. S.A. and J.A. are well-known contributors to the plant immune system, promoting disease resistance and defense (Bürger and Chory, 2019). In *Arabidopsis*, TGA genes directly activate the expression of SA-responsive genes *RBOHD* and *RBOHF*, whereas RipAB, an effector of *Ralstonia solanacearum*, disrupts SA signaling by inhibiting TGA activity to facilitate infection (Qi et al., 2022). After SA treatment, most *CsTGA* genes were markedly upregulated, whereas only *CsTGA2*, *CsTGA4*, *CsTGA6*, and *CsTGA7* were significantly upregulated after JA treatment. Notably, *CsTGA1* exhibited marked upregulation after SA treatment (35.08-fold) but considerable downregulation after JA treatment (0.04-fold), indicating an antagonistic function in SA- and JA-dependent immune responses.

### *CsTGA* genes are involved in abiotic stresses responses

In recent years, numerous studies have reported the roles of TGA TFs in plant responses to abiotic stresses. *GmTGA17* confers drought and salt stress tolerance in transgenic soybean plants (Li et al., 2019a). In Arabidopsis, multiple class II TGA TFs function as key regulators of genes controlling reactive oxygen species (ROS) levels during UV-B stress (Herrera-Vásquez et al., 2021). qRT-PCR analysis indicated that *CsTGA1*, *CsTGA2*, *CsTGA3*, and *CsTGA6* were significantly downregulated, while the remaining three *CsTGA* genes were dramatically upregulated under cold treatment (**Figure 5B**). Interestingly, the expression patterns of *CsTGA* genes under cold treatment were consistent with those under ABA treatment, except for *CsTGA4*. Previous studies have shown that plant tolerance to low-temperature stress is regulated by complex phytohormone signaling pathways and metabolic processes, especially involving the stress-responsive phytohormone ABA (Huang et al., 2008). These findings suggest that *CsTGA* genes may play a critical role in the low-temperature response pathway of *C*. *sinensis*, which is dependent on the ABA signaling pathway. Noteworthy to emphasize that six *CsTGA* genes were markedly upregulated under dehydration treatment (except for *CsTGA4*). In contrast, the same six genes were significantly downregulated under salt treatment. Both dehydration and salt stress are known to induce osmotic injury to plants (Zhao et al., 2023b). However, the secondary effects of dehydration and salt stress are more complex, including oxidative stress, damage to cellular components (e.g., membrane lipids, proteins, and nucleic acids), and metabolic disorders (Zhu, 2016; Waadt et al., 2022). Thus, the opposite expression trends of *CsTGA* genes under dehydration and salt stress may reflect their involvement in distinct signaling mechanisms for these stresses. In summary, all *CsTGA* genes are involved in the abiotic stress responses of *C*. *sinensis*, including cold, dehydration, and salt stresses. However, further validation is needed to elucidate the specific functions of *CsTGA* genes under abiotic stress.

### CsTGA7 localizes in the nucleus and exhibits transcriptional activation activity

Numerous studies have demonstrated that the subcellular localization and transcriptional activation activity of TFs are critical features for their biological functions (Geng and Liu, 2018; Dai et al., 2023). These characteristics collectively determine the central role of TFs in cellular signaling and gene regulatory networks. Our findings reveal that CsTGA7 is specifically localized in the nucleus (**Figures 7A, B**), which is consistent with the functional properties of TFs. Nuclear localization enables TFs to directly interact with chromatin and regulate gene expression (Millar et al., 2009). Furthermore, yeast system experiments confirmed that CsTGA7 possessed transcriptional activation activity, with its N-terminal region being essential for this function (**Figure 7C**). Transcriptional activation activity allows TFs to recruit transcriptional machinery or co-activators, thereby initiating or enhancing the transcription of target genes (He et al., 2024). We note that the N-terminal region contains a conserved bZIP domain, suggesting its potential role in the transcriptional activation function of TGA proteins.

## Conclusions

Seven *CsTGA* genes were identified and characterized in *C*. *sinensis* genome. Gene structure and phylogenetic analysis indicated that *CsTGA* family members were highly conserved, with *CsTGA7* potentially occupying a basal position in the evolutionary history of the family. qRT-PCR analysis showed that *CsTGA* genes exhibited diverse response patterns under phytohormone and abiotic stress treatments. In-depth analyses indicated that CsTGA7, which exhibited the most robust response to various stress treatments, was specifically localized in the nucleus and possessed transcriptional activation activity, consistent with the typical characteristics of TFs.

## Data Availability

The datasets presented in this study can be found in online repositories. The names of the repository/repositories and accession number(s) can be found in the article/**Supplementary Material**.

## Supplementary material

Supplementary Table 1 Accession numbers of TGA proteins from databases.

Supplementary Table 2 Primer sequences used in this study.

Supplementary Table 3 Nomenclature and key features of *CsTGA* genes.

Supplementary Table 4 Conserved motifs in CsTGA proteins.

Supplementary Table 5 List of stress-responsive *cis*-acting elements identified in the 2.5 kb upstream regions of *CsTGA* genes.

Supplementary Table 6 qRT-PCR expression values of *CsTGA* genes under multiple phytohormone treatments.

Supplementary Table 7 qRT-PCR expression values of CsTGA genes under multiple abiotic stresses.

Supplementary Figure 1 Composition of conserved domains in CsTGA proteins.
