# Supplementary figures and images for: Genome-wide characterization and expression profiling of the TGA gene family in sweet orange (Citrus sinensis) reveal CsTGA7 responses to multiple phytohormones and abiotic stresses
Source: Front Plant Sci. 2025 Feb 25;16:1530242. doi: 10.3389/fpls.2025.1530242 (PMC11893830; doi:10.3389/fpls.2025.1530242)

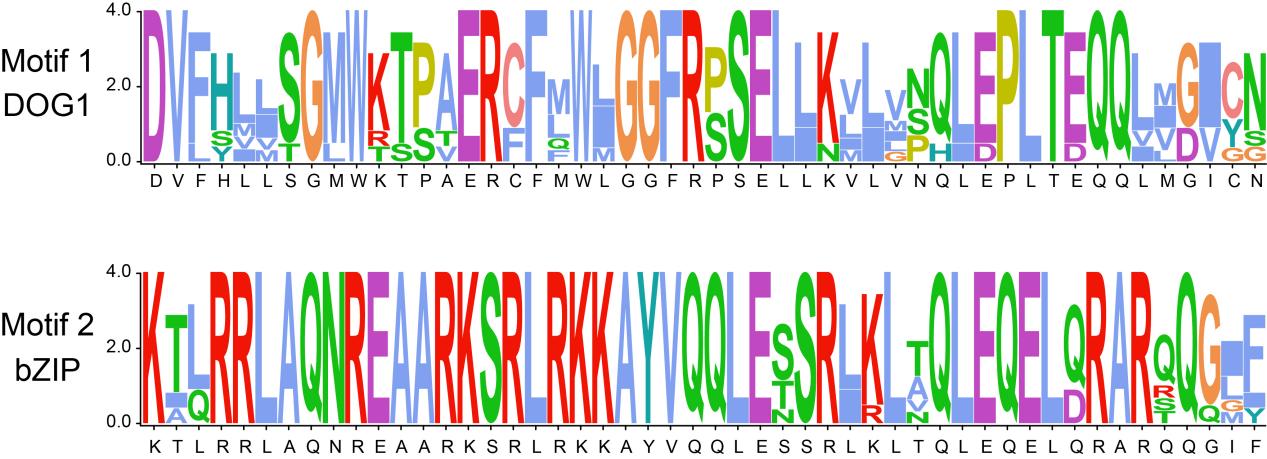


**Figure S1. Composition of conserved domains in CsTGA proteins.**

Supplement: Supplementary Figure 1 — Composition of conserved domains in CsTGA proteins. [file Table2.docx]
